# Supplementary material for: Modelling the modulation of cortical Up-Down state switching by astrocytes
Source: PLoS Comput Biol. 2022 Jul 21;18(7):e1010296. doi: 10.1371/journal.pcbi.1010296 (PMC9345492; doi:10.1371/journal.pcbi.1010296)
Supplement: S1 Text — (PDF) [file pcbi.1010296.s001.pdf]

Modelling the modulation of cortical Up-Down state switching by astrocytes

Lisa Blum Moyse[1,2], Hugues Berry[1,2,*]

1 Inria, Villeurbanne, France
2 LIRIS UMR5205, University of Lyon, Villeurbanne, France

* hugues.berry@inria.fr

Supporting Information

S1 Text. Fixed points and linear stability analyses: rate model

Noiseless model. We start with the rate model defined by equations (1) to (6) and first neglect the external noisy input. In this case, the nullclines of the system are given by

$$r_E = g_E[I_E - a - \theta_E]_+ \tag{S1.1}$$

$$r_I = g_I[I_I - \theta_I]_+ \tag{S1.2}$$

$$r_A = g_A[I_A - \theta_A]_+ \tag{S1.3}$$

$$a = \beta r_E \tag{S1.4}$$

Note that because of the rectification functions in equations (S1.1) to (S1.3), the values of the rates at a fixed point cannot be negative. The rate model equations (1) to (6) being a piecewise-smooth system, a rigorous analysis of the stability of its fixed points would require dedicated analysis methods [1]. Here, we leave this analysis for further work and assume that all fixed points remain far from the switching manifolds where the arguments of the rectification functions change signs and proceed to linear stability analysis in each of the respective regions.

A Down or silent state can be characterized as a fixed-point where both neuronal populations are silent, i.e. $r_E = r_I = 0$ spks/s. Eq (S1.4) means that adaptation a also vanishes in such a Down state. The rectification functions of equations (S1.1) and (S1.2) impose $\theta_E \geq 0$ and $\theta_I \geq 0$ for the Down fixed-point to exist. Indeed $\theta_E < 0$ would mean from Eq (S1.1) that $r_A < 0$ at the fixed-point (since $r_E = 0$ spks/s), which is not compatible with Eq (S1.3). Hence the Down state exists only if $\theta_E \geq 0$ or $\theta_I \geq 0$. If, in addition $\theta_A \geq 0$, the Down state is $(r_E, r_I, r_A, a) = (0, 0, 0, 0)$. Assuming that all the rectification functions in Eq (1), Eq (2) and Eq (5) vanish in the neighborhood of the fixed point, we find that this Down state is stable by linear stability analysis (eigenvalues of the Jacobian:$\{-1/\tau_E, -1/\tau_I, -1/\tau_A, -1/\tau_a\}$).

In the case $\theta_A < 0$ (still with $\theta_I \geq 0$ and $\theta_E \geq 0$), we assume that the argument of the rectification function in Eq (5) is strictly positive, while the rectification functions in equations (1) and (2) vanish. The nullcline for r_A, Eq (S1.3) then becomes $r_A = g_A(J_{AA} r_A - \theta_A)$. Therefore, there still is a positive Down fixed-point $(r_E, r_I, r_A, a) = (0, 0, -g_A\theta_A/(1 - g_A J_{AA}), 0)$ but only for:

$$\theta_E > -g_A J_{EA} \theta_A/(1 - g_A J_{AA}), \tag{S1.5}$$

as well as $\theta_I > -g_A J_{IA}\theta_A/(1-g_A J_{AA})$ and $g_A J_{AA} < 1$. Close to this fixed-point, the Jacobian matrix reads

$$\begin{pmatrix} -1/\tau_E & 0 & 0 & 0 \\ 0 & -1/\tau_I & 0 & 0 \\ 0 & 0 & (g_A J_{AA}-1)/\tau_A & 0 \\ \beta & 0 & 0 & -1/\tau_a \end{pmatrix} \tag{S1.6}$$

so stability is granted whenever $g_A J_{AA} < 1$, i.e as soon as the equilibrium value for r_A exists.

To find an Up state fixed-point with non-zero rates we follow [2] and substitute the value of the adaptation at equilibrium $a = \beta r_E$, assuming that the arguments of all the rectification functions of Eq (1), Eq (2) and Eq (5) are strictly positive. This yields:

$$r_E = \frac{h_E\theta_E + f_{EI}\theta_I + f_{EA}\theta_A}{|M|}, \tag{S1.7}$$

$$r_I = \frac{f_{IE}\theta_E + (h_I - \beta J'_{AA})\theta_I + (f_{IA} + J_{IA}\beta)\theta_A}{|M|} \tag{S1.8}$$

and

$$r_A = \frac{f_{AE}\theta_E + (f_{AI} + J_{AI}\beta)\theta_I + (h_A - \beta J'_{II})\theta_A}{|M|} \tag{S1.9}$$

with

$$J'_{XY} = J_{XY} - \frac{1}{g_X}, \tag{S1.10}$$

$$h_X = J'_{YY}J'_{ZZ} - J_{YZ}J_{ZY}, \tag{S1.11}$$

$$f_{XY} = J_{XZ}J_{ZY} - J_{XY}J'_{ZZ}, \tag{S1.12}$$

and

$$|M| = J_{AE}f_{EA} + J_{AI}(f_{IA} + J_{IA}\beta) + J'_{AA}(h_A - \beta J'_{II}) \tag{S1.13}$$

One condition for the UP state fixed-point to exist is that the right hand side of Eq (S1.7), Eq (S1.8) and Eq (S1.9) are positive. Given our reference parameters (table 1), the condition on r_I, i.e., $r_I > 0$ is the most restrictive condition. In other words, when θ_E and β are varied, Eq (S1.8) is the first one to become positive. Moreover, with our reference parameters, it turns out that the determinant $|M| < 0$. The condition for the existence of the Up state fixed-point thus becomes

$$\beta < \frac{f_{IE}\theta_E + h_I\theta_I + f_{IA}\theta_A}{J'_{AA}\theta_I - J_{IA}\theta_A}. \tag{S1.14}$$

since $J'_{AA}\theta_I - J_{IA}\theta_A < 0$ with our reference parameters (table 1).

Considering that in the neighborhood of the Up fixed-point, all the rectification functions of the model are positive, the Jacobian matrix reads

$$\begin{pmatrix} \frac{-1+g_E(J_{EE}-\beta)}{\tau_E} & \frac{g_E J_{EI}}{\tau_E} & \frac{g_E J_{EA}}{\tau_E} \\ \frac{g_I J_{IE}}{\tau_I} & \frac{-1+g_I J_{II}}{\tau_I} & \frac{g_I J_{IA}}{\tau_I} \\ \frac{g_A J_{AE}}{\tau_A} & \frac{g_A J_{AI}}{\tau_A} & \frac{-1+g_A J_{AA}}{\tau_A} \end{pmatrix}.$$

It is possible to obtain analytical expressions for the eigenvalues of this matrix, however these expressions are too complex to be really useful, even to determine their signs. We therefore estimated their values numerically to explore stability of the Up fixed-point, with numerical estimation of the sign of the real part of the eigenvalues of the matrix. Note finally that in regions where both the Up state and the Down state fixed-points are stable, the existence of a third, unstable and intermediate fixed-point can be evidenced by numerical analysis.

The effect of noise on the model. The addition of noise however complicates the above picture. In particular, spontaneous Up-Down transitions can occur even in the region where the Down state is stable and the Up state unstable, through a dynamical regime whereby noise triggers the Down to Up switches and adaptation triggers the reverse Up to Down transitions. In [2], it is proposed that this subregion can be estimated to start when the Up state is unstable under the sole influence of adaptation, a condition that can be deduced from Eq (S1.14) with $\beta = 0$:

$$\theta_E < -\frac{h_I \theta_I + f_{IA}\theta_A}{f_{IE}} \tag{S1.15}$$

Likewise, the symmetrical regime exists in the region where the Up state is stable and the Down state unstable, for which Up-to-Down transitions are triggered by noise and Down-to-Up switches by adaptation. Ref. [2] proposes to delineate this region by the situation where adaptation in the Up-state is large enough to counterbalance the effect of θ_E, i.e. $\beta r_E + \theta_E > 0$, where r_E is given by Eq (S1.7), which yields

$$\beta > \frac{-\theta_E (J_{AE} f_{EA} + J_{AI} f_{IA} + J'_{AA} h_A)}{f_{EI}\theta_I + f_{EA}\theta_A + (h_E - J'_{AA} J'_{II} - J_{AI} J_{IA})\theta_E} \tag{S1.16}$$

References

1. di Bernardo M, Budd C, Champneys A, Kowalczyk P. Piecewise-smooth dynamical systems: theory and applications. Applied Mathematical Sciences. Springer; 2008.

2. Jercog D, Roxin A, Barthó P, Luczak A, Compte A, de la Rocha J. UP-DOWN cortical dynamics reflect state transitions in a bistable network. eLife. 2017;6. doi:10.7554/eLife.22425.
