# Supplementary material for: Modelling the modulation of cortical Up-Down state switching by astrocytes
Source: PLoS Comput Biol. 2022 Jul 21;18(7):e1010296. doi: 10.1371/journal.pcbi.1010296 (PMC9345492; doi:10.1371/journal.pcbi.1010296)
Supplement: S2 Text — (PDF) [file pcbi.1010296.s002.pdf]

Modelling the modulation of cortical Up-Down state switching by astrocytes

Lisa Blum Moyse[1,2], Hugues Berry[1,2,*]

1 Inria, Villeurbanne, France
2 LIRIS UMR5205, University of Lyon, Villeurbanne, France

* hugues.berry@inria.fr

Supporting Information

S2 Text. Fixed points and linear stability analyses: spiking model

Analysis of the spiking model defined by equations (7) to (13) was carried out as follows. The nullclines of the population averaged rates are obtained from the equilibrium firing rate ($r_{X,0}$), which is given by the self consistent mean-field equation [1]:

$$r_{X,0} = \frac{1}{\tau_X}\left[\int_0^\infty \frac{dy}{y} e^{-y^2}\left(e^{2yy_t^X} - e^{2yy_r^X}\right)\right]^{-1} \tag{S1.17}$$

with

$$y_r^X = \frac{V_r - I_{X,0}}{\sigma_X} \qquad y_t^X = \frac{\theta_X - I_{X,0}}{\sigma_X} \tag{S1.18}$$

and with the currents:

$$I_{E,0} = V_{L,E} + I_{rec,E} - K_a \beta r_{E,0} \tag{S1.19}$$

$$I_{I,0} = V_{L,I} + I_{rec,I} \tag{S1.20}$$

$$I_{A,0} = V_{L,A} + I_{rec,A} \tag{S1.21}$$

$$I_{rec,X} = C_{XE} J_{XE} r_{E,0} \underline{\tau_E} + C_{XI} J_{XI} r_{I,0} \underline{\tau_I} + C_{XA} J_{XA} r_{A,0} \underline{\tau_A} \tag{S1.22}$$

where C_{XY} is the connectivity (0.1 for astrocyte to neuron connection, 0.5 for neuron to astrocytes, 1.0 otherwise). Note that we replaced $I_{a,0} = \beta r_{E,0}$. These self-consistent equations are solved by finding the intersection between the right and the left side of equation (S1.17). The fixed points are the intersections of the three surfaces $r_{E,0}$, $r_{I,0}$ and $r_{A,0}$. The stability of these fixed points is determined with the sign of the eigenvalue λ [1–3]. Solving the system

$$\begin{cases} \delta r_E = F_{EE}(\lambda)\delta r_E + F_{EI}(\lambda)\delta r_I + F_{EA}(\lambda)\delta r_A \\ \delta r_I = F_{IE}(\lambda)\delta r_E + F_{II}(\lambda)\delta r_I + F_{IA}(\lambda)\delta r_A \\ \delta r_A = F_{AE}(\lambda)\delta r_E + F_{AI}(\lambda)\delta r_I + F_{AA}(\lambda)\delta r_A \end{cases} \tag{S1.23}$$

for the perturbations around the fixed-point, δr_E, δr_I and δr_A, one gets the condition (omitting the dependences on λ for readability):

$$\begin{gathered} (F_{EE}-1)(F_{II}-1)(F_{AA}-1) + F_{EI}F_{IA}F_{AE} + F_{EA}F_{IE}F_{AI} \\ -(F_{EE}-1)F_{IA}F_{AI} - F_{EI}F_{IE}(F_{AA}-1) - F_{EA}(F_{II}-1)F_{AE} = 0 \end{gathered} \tag{S1.24}$$

with

$$F_{XY}(\lambda) = J_{XY} R_X(\lambda) S_Y(\lambda) \tag{S1.25}$$

and the synaptic response function $S_Y(\lambda)$

$$S_Y(\lambda) = \frac{e^{-\lambda d^Y}}{(1+\lambda\tau_r^Y)(1+\lambda\tau_d^Y)} \tag{S1.26}$$

In this equation, $R_X(\lambda)$ is the neuronal response function defined as

$$R_X(\lambda) = \frac{r_{X,0}}{\sigma_X(1+\lambda\tau_X)} \frac{\frac{\partial U}{\partial y}(y_t^X, \lambda\tau_X) - \frac{\partial U}{\partial y}(y_r^X, \lambda\tau_X)}{U(y_t^X, \lambda\tau_X) - U(y_r^X, \lambda\tau_X)} \tag{S1.27}$$

with

$$U(y,\lambda) = \frac{e^{y^2}}{\Gamma\left[\frac{1+\lambda}{2}\right]} M\left(\frac{1-\lambda}{2}, \frac{1}{2}, -y^2\right) + \frac{2ye^{y^2}}{\Gamma\left[\frac{\lambda}{2}\right]} M\left(1-\frac{\lambda}{2}, \frac{3}{2}, -y^2\right) \tag{S1.28}$$

with M is a confluent hypergeometric function. Stability is assessed by solving Eq (S1.24) for λ numerically. The fixed point is stable when its real part is negative.

References

1. Brunel N, Hakim V. Fast Global Oscillations in Networks of Integrate-and-Fire Neurons with Low Firing Rates. Neural Computation. 1999;11:1621–1671. doi:10.1162/089976699300016179.

2. Roxin A, Compte A. Oscillations in the bistable regime of neuronal networks. Physical review E. 2016;94:012410. doi:10.1103/PhysRevE.94.012410.

3. Ledoux E, Brunel N. Dynamics of networks of excitatory and inhibitory neurons in response to time-dependent inputs. Frontiers in Computational Neuroscience. 2011;5. doi:10.3389/fncom.2011.00025.
