# Supplementary figures and images for: Modelling the modulation of cortical Up-Down state switching by astrocytes
Source: PLoS Comput Biol. 2022 Jul 21;18(7):e1010296. doi: 10.1371/journal.pcbi.1010296 (PMC9345492; doi:10.1371/journal.pcbi.1010296)

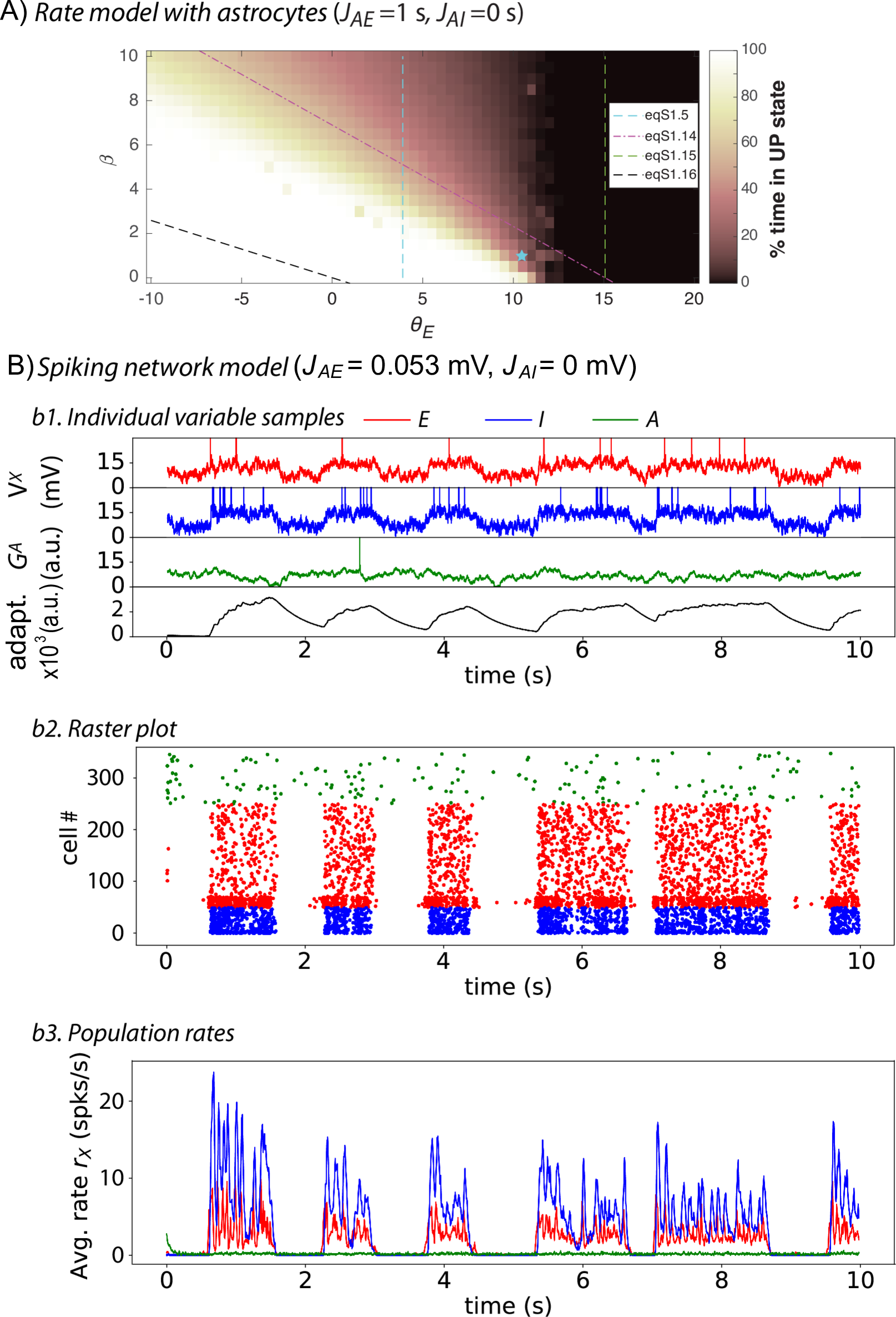

Supplement: S1 Fig — Results of the rate model of Eqs (1) to (6) (A) or the spiking network model of Eqs (7) to (13). (B) obtained in the presence of astrocytes, but with JAI = 0 mV, i.e. with no direct effect of inhibitory neurons on astrocytic gliotransmitter release. All parameters were as indicated in Tables 1 or 2, except for the value of JAI that was set to 0. Refer to Figs 3C and 5 for the color-codes and parameters of panels (A) and (B), respectively. (TIF) [file pcbi.1010296.s003.tif]
