# Supplementary material for: Modelling the modulation of cortical Up-Down state switching by astrocytes
Source: PLoS Comput Biol. 2022 Jul 21;18(7):e1010296. doi: 10.1371/journal.pcbi.1010296 (PMC9345492; doi:10.1371/journal.pcbi.1010296)
Supplement: Attachment — Submitted filename: PointbyPointReply.docx [file pcbi.1010296.s004.docx]

## “Modelling the modulation of cortical Up-Down state switching by astrocytes” by Blum-Moyse and Berry

## Point by point reply to the reviewers

We first want to thank the four reviewers for their analysis of our manuscript, and their insightful suggestions that have helped us improve our study. We present below our detailed answers to the issues they raised.

The comments from the reviewers are in light green and our responses are in black font. We also include the corresponding text modifications, when appropriate, in blue. In the revised manuscript itself, modifications compared to the previous version of the manuscript are also in blue. We modified the title of a figure to make it shorter, in orange.

**Reviewer #1:**

This work adds to previous theoretical studies investigating how Up-Down cortical dynamics is triggered by the interplay between an intrinsic activity-dependent negative feedback of the firing rate and an external input to the network. Here, the authors studied the modulation by astrocytes with mathematical models. Their work is based on an existing compartment model on Up-Down cortical dynamics, which was extended by the authors to study the effect of astrocytes. Both a simple rate and an otherwise equal stochastic modelling framework were set up. Necessary core information of the model and its parameters as well as assumptions are described in the main text and more details in the supplement, which also provides more insights to the applied fixed-point and linear stability analysis. Comparisons of structural model differences and analysis of the parameter space indicate that the presence of astrocytes can induce the emergence of Up-Down cortical dynamics and can provide more clear differences between firing rates in Up and Down phases than the model with no astrocytes. The results provide useful insights into the dynamic regime of the model, indicating that gliotransmission between astrocytes and neurons allows Up-Down cortical dynamics to occur under a broader range of conditions. Finally, the general results are further confirmed by robustness tests, e.g. relaxing the condition that J_AI > 0, which is good modelling practice.

Overall, the manuscript demonstrates sound modeling work, is well structured and is written in plain language understandable to nonspecialists. I am happy to report that I only have a few small comments.

We thank the reviewer for her/his very positive appreciation of the manuscript. We also thank her/him for the minor comments that we reply to below.

Minor comments:

Figure 1: the latin “a” is shown instead of the greek letter alpha, which is referenced in the figure caption and model equations in the text.

We thank reviewer#1 for pointing out this source of ambiguity. We have corrected figure 1 accordingly.

Lines (457 to 466) 467 to 474: Such a comparison to a specific empirical study is nice, but it should be clear whether some sort of optimization of parameter values has been used to get this agreement. Multi-objective optimization can be used to define a parameter set that is providing the best match. It would be interesting to see whether one, both or none of the models with and without astrocytes can be parameterized in a way providing a nearly perfect match to these empirical estimates.

We thank the reviewer for this comment. Actually, we did not use a specific parameter calibration methodology in the manuscript. The main reason for this is that the available experimental data exhibit quite a range of variability. For instance, in Jercog et al, 2017, the experimental quantities used in the discussion to compare with our model (Up or Down phase length, CV of the rates) vary quite a lot from one repetition of the experiment to the other. Over the seven repetitions of the same experiments which results are shown in their Figure 2A and B, the mean duration of the Up phases varies from 0.24s to 0.73s, for example. With other experimentalists, using other experimental setups, and recording on different cortical regions, the variability would probably be even larger.

Therefore, parameter optimization would at best have allowed to match one specific repetition of a given experiment but it is not clear what we would have learned from it. Instead, we have chosen to set the initial guess for the parameters to rough estimates from the literature when available. For instance, the quantification of propagation delays of calcium waves in 3D astrocytes made in Bindocci et al, 2017 sets an order of magnitude of around 1 to 2 sec for the maximal delay *d*_max_ of astrocytes. For the other parameters, we used bifurcation studies like those shown in the manuscript to locate regions of the parameter space in which model simulations are approximately in agreement with the variation range of the main experimental quantities of Jercog et al, 2017.

***Changes made:***

We have added a new subsection of the Methods section to explain the strategy we used to fix parameter values (section 2.3 “Parameter estimation”). We also have added a piece of text to the discussion paragraph dedicated to the comparison of the model results with the experimental results to highlight this fact.

- Line 189: “**2.3. Parameter estimation**. The experimental data available for parameter estimation of the two models above exhibit strong variability. In Jercog et al, 2017, for instance, the numbers used to quantify the experimental measurements (distribution of duration of Up or Down phases, CV of the firing rates) vary significantly from one repetition of the experiment to the other. Over the seven repetitions of the same experiments (their Figure 2A and B), the mean duration of the Up phases varies from 0.24s to 0.73s, for example. With other experimentalists, using other experimental setups, and recording on different cortical regions, the variability would probably be even larger. Therefore, the classical methodologies for parameter estimation would at best allow to match one specific repetition of a given experiment, where our objective here is to get a more generic overview of this system. Therefore, we have opted for an ad hoc method to set the values of the parameters. When available, we have set the initial guess for the parameters to rough estimates from the literature. For instance, the quantification of propagation delays of calcium waves in 3D astrocytes made in Bindocci et al, 2017 sets an order of magnitude of around 1 to 2 sec for the maximal delay of astrocytes $d^A_{\max}$. For the other parameters, we used bifurcation studies like those shown below to locate regions of the parameter space in which model simulations are approximately in agreement with the variation range of the main experimental quantities of Jercog et al, 2017. The results are given in table 1 and table 2.”
- Line 424 (Discussion): As explained in section 2.3, parameter values were set so that the model simulations exhibit behaviors similar to the experiments of Jercog et al, 2017. We now give a more detailed account of the match between model and data.

**Reviewer #2:**

In this paper, a theoretical framework is provided to test scenarios and hypotheses on the modulation of Up-Down dynamics by gliotransmission from astrocytes. Three populations’ cells are considered which are interconnected by gliotransmission events from neurons to astrocytes and back. Two models are derived for this three-population system: a rate model and a stochastic spiking neural network. Even the paper is well written and involves an interesting idea; however, it requires some corrections. The authors must arrange their paper once again using following comments:

We thank the reviewer for his/her positive opinion on our manuscript and for the insightful suggestions. We took them all into account in the revised version, that, we think, has been significantly improved.

1) Concise the abstract providing quantitative analysis as well as main features of the problem. Include other detail in discussion section.

We agree with this comment and we are sorry that the abstract was indeed too long in the first version. We have completely rewritten it in the revised version. We hope that this new version will be more concise and more straight-to-the-fact. Note that we have also added an “author summary” as per instructions of *PLoS Computational Biology*.

***Changes made:***

- The abstract has been rewritten.

- Addition of the Author summary

2) Difficult wording is used throughout the manuscript. There is no need for ambiguous wordings. Just make it simple and readable.

We thank the reviewer for this comment and we are sorry that the language of the initial version of manuscript was clearly perfectible. We have taken great care to revise the language of the manuscript with the objective to make it simpler and more readable. We hope the reviewer will find the revised version improved in this aspect too.

***Changes made:***

- The revised version shows in green all the modifications that we have made in the text. As can be seen from inspection of the overall text color, we have simplified a great number of convoluted wording occurrences.

3) Is there any base to assume that each population receives a fluctuating external input?

Available experimental evidence suggests that Up-Down regimes in the cerebral cortex can be strongly coupled to the activity of subcortical or limbic areas, many of which are known to oscillate at low frequencies. The fluctuating external input emulates such a type of external input. To our knowledge, there are no a priori reasons to consider that these external inputs are restricted to a subtype of brain cells either excitatory neurons, inhibitory neurons or astrocytes. In our models, an oscillatory external input was thus fed to all cell populations.

***Changes made:***

- We have tried to make this point clearer. We have first introduced some explanation about the origin of the fluctuating input in the rate model.

Line 75: “Several experimental studies have evidenced that oscillatory inputs coming from the thalamus or other subcortical areas have a strong influence on cortical Up-Down dynamics (Rigas and Castro-Alamancos, 2007; David et al., 2013; Lemieux et al., 2014). The external input ξ_X_ emulates such an oscillatory external input, as an independent Ornstein-Uhlenbeck process (see Jercog et al, 2017 for details).”

- We have also insisted that we have no reason a priori to restrict this input to any of the three populations of the model.

Line 107: “To our knowledge, there are no a priori reasons to consider that external inputs to the cortical network under study are restricted to a given subtype of brain cells, either excitatory neurons, inhibitory neurons or astrocytes. Therefore, we fed an oscillatory external input was to all cell populations, including astrocytes (variable ξ_X_ above).”

4) What is new in the model equations (1) to (4) and why these are considered? Discuss in the paper.

The main novelties in model equations (1) to (4) are *i*) to introduce the impact of astrocytes in neuronal networks with Up-Down dynamics and *ii*) to account for the influence of astrocytes using a rate equation with a similar mathematical structure as the firing rate equation of the neurons. Our motivation was to introduce the impact of astrocyte activity since recent experimental results indicate that these non-neuronal cells control the emergence of Up-Down dynamics in the surrounding neuronal network. Modelling the gliotransmitter release activity of astrocytes using a rate equation with a similar mathematical structure to the firing rate equation of the neurons enabled us to preserve the mathematical tractability of the model. We have modified the discussion section with the objective to make this point clearer.

***Changes made:***

- Line 424: “These results motivated us to propose our rate model equations (1) to (6). The main novelties here are i) to introduce the impacts of astrocytes in the dynamics of neuronal networks in the Up-Down regime and ii) to account for the influence of astrocytes using a rate equation with a mathematical structure similar to the firing rate equation of the neurons. Modelling the gliotransmitter release activity of astrocytes using a rate equation similar to the firing rate equation of the neurons enabled us to preserve the mathematical tractability of the model.”

5) Correlate results of three-population systems with the earlier ones (even in limiting cases).

Comparison with earlier models is uneasy because those earlier models did not incorporate our main populations of interest. Indeed, our three-population system provides information on the influence of the third population (the astrocytes) that cannot be obtained with earlier ones, that did not account for the effect of astrocytes. This is the main contribution of our new model, and the main justification for its novelty. We have tried to make this clearer in the revised version, by adding a new paragraph on this point.

***Changes made:***

- Line 437: “In our numerical simulations, the addition of gliotransmission from astrocytes was sufficient to transform a neural network prepared in the Down, silent state into a dynamical regime of spontaneous alternations between Up and Down states. The inclusion of astrocytes in our model therefore provided us with the opportunity to explore how astrocytes alter the dynamics of the neuronal firing rates in a way that switches them to the Up-Down alternation regime. A major conclusion from our model is that gliotransmission probably does not have a drastic effect on the underlying dynamics of the network. Adding gliotransmission does not modify the number nor the type of the observed bifurcations, it only alters the values of the parameters at which these bifurcations occur. As a result, gliotransmission can transform a silent neural network model into a network exhibiting Up-Down dynamics, with no change of the neuron-related parameters, and no alterations of the neural mechanisms that control the transitions between Up and Down phases. Moreover, our model suggests that the fundamental differences of signal integration in neurons versus astrocytes may be crucial in the emergence of Up-Doc regimes. In particular, the signaling delay in our spiking network model was kept three orders of magnitude larger in astrocytes compared to neurons i.e., seconds versus milliseconds. This difference of timescales turned out to be crucial for the network dynamics illustrated in fig. 5 where a stationary background of astrocytic gliotransmission events triggers spontaneous transitions between synchronized Up and Down phases of neuronal firing.”

**Reviewer #3:**

I read the manuscript entitled " Modelling the modulation of cortical Up-Down state switching by astrocytes" with great interest. It has been a comprehensive study, and I think it has valuable content.

We thank the reviewer for this very positive assessment of our work.

However, the following significant corrections seem necessary to improve the scientific level of the article.

We also thank reviewer#3 for his/her corrections above that have all been take into account in the revised version. We think this has significantly increased the quality of our paper.

1- Please rewrite the abstract section. It has a lot of unnecessary descriptions.

We are sorry that the abstract was indeed too long in the first version. We have completely rewritten it in the revised version. We hope that this new version will be more concise and more straight-to-the-fact. Note that we have also added an “author summary” as per instructions of *PLoS Computational Biology*.

***Changes made:***

- The abstract has been rewritten.

- Addition of the Author summary

2- Please explain more clearly the limitation and future work.

The initial version already presented the limitation of our work in the discussion section but we agreed with the reviewer that this was not very clearly written, in particular because the discussions about limitations and corresponding future work were somewhat spread over the whole section. In the revised version, we have reshaped the discussion section to cluster all the remarks related to limitation and future work in the same place, i.e. under the same paragraph.

***Changes made:***

- Line 457: A new paragraph now focus on limitation and future work.

“The main limitation of our models are the simplification assumptions that we made to express the impact of astrocytes on the neural network model. The modeling literature proposes mathematical descriptions of the process of gliotransmitter release from astrocytes that are much more complex or accurate than the simple phenomenological expressions used here, see e.g., De Pitta and Berry (2019) for a recent account. However the price to pay for the added complexity would be a restriction of the available mathematical understanding of the system dynamics. Future numerical simulation works will be needed to assess whether the inclusion of such more complex descriptions comes with changes of the main conclusions of the present study. We also adopted the modelling choice made by Jercog et al. (2017) for their spiking model where the synapse dynamics are modelled using a single population variable, integrating the spikes emitted by the whole population into a single variable that can then be fedback to the other cells. This choice limits the range of modelling exploration regarding connectivity. It forbids models where the inputs received by an astrocyte is restricted to a subset of the neurons or, conversely, those where gliotransmission from an astrocyte targets only a subset of the synapses of a neuron. On the other hand, though, this modelling choice greatly facilitates theoretical (mean-field) analysis of the stochastic network model. We leave for future works the study of models that would incorporate the main ingredients of our models above, but with real individual synapses and / or more realistic sparse neuron-neuron connectivity (Tartaglia and Brunel, 2017). This would make it possible to associate a spatial embedding to the network thus enabling the study of slow wave propagation (Cakan et al., 2021) or to compare Up-Down regimes during sleep with those observed during anasthesia (Torao-Angosto et al., 2021).”

3- The discussions should highlight why the proposed method is providing good results. The comparative study from the recently proposed approach is missing.

Obviously, our three-population system provides information on the influence of the third population (the astrocytes) that cannot be obtained with earlier ones, that did not account for the effect of astrocytes. This is the main contribution of our new model, and the main justification for its novelty. We have tried to make this clearer in the revised version, by adding a new paragraph on this point.

***Changes made:***

- Line 437: “In our numerical simulations, the addition of gliotransmission from astrocytes was sufficient to transform a neural network prepared in the Down, silent state into a dynamical regime of spontaneous alternations between Up and Down states. The inclusion of astrocytes in our model therefore provided us with the opportunity to explore how astrocytes alter the dynamics of the neuronal firing rates in a way that switches them to the Up-Down alternation regime. A major conclusion from our model is that gliotransmission probably does not have a drastic effect on the underlying dynamics of the network. Adding gliotransmission does not modify the number nor the type of the observed bifurcations, it only alters the values of the parameters at which these bifurcations occur. As a result, gliotransmission can transform a silent neural network model into a network exhibiting Up-Down dynamics, with no change of the neuron-related parameters, and no alterations of the neural mechanisms that control the transitions between Up and Down phases. Moreover, our model suggests that the fundamental differences of signal integration in neurons versus astrocytes may be crucial in the emergence of Up-Doc regimes. In particular, the signaling delay in our spiking network model was kept three orders of magnitude larger in astrocytes compared to neurons i.e., seconds versus milliseconds. This difference of timescales turned out to be crucial for the network dynamics illustrated in fig. 5 where a stationary background of astrocytic gliotransmission events triggers spontaneous transitions between synchronized Up and Down phases of neuronal firing.”

4- How did the authors assume the connectivity rules?

Our choice of connectivity rule was done in order to account for generic experimental observations such as the fraction of synapses contacted by an astrocyte (tripartite synapses) or the extent of astrocyte-to-astrocyte connectivity.

We have explained our choices more clearly in the revised version.

***Changes made:***

- Line 141: “For the simulations of the spiking model, we assumed the following connectivity rules:

- full connectivity for neuron-to-neuron connections and for astrocyte-to-astrocyte connections. The latter emulates the organization of astrocytes as a syncytium. This biological concept corresponds to the idea that all astrocytes of a local region are somehow interconnected together into single functional network (Verkhratsky and Nedergaard, 2018).

- only a fraction (10 %) of E or I neurons are subjected to gliotransmission from the astrocytes. These neurons are chosen at random (uniform distribution).

- 50 % of the astrocytes, chosen uniformly at random, receive inputs from the E or I neurons.

The latter two connectivity rules account from the observation that only part of the synapses of a given brain region are tripartite synapses contacted by astrocytes. The exact fraction seems to be quite variable from one region to the other, from 10% to 90% (Verkhratsky and Nedergaard, 2018). Therefore, our choice corresponds to a lower range of parameters.

5- The "Discussion" section should be added in a more highlighting, argumentative way.

We agree with the reviewer that the discussion section of the initial version of the manuscript lacked clarity and focus. As can be seen from the amount of new, green, text in the revised version we have extensively reshaped its content and rewritten it, with the objective of increased emphasis.

***Changes made:***

- The discussion section has been thoroughly reworked with the objective of increased focus and emphasis.

Please note that the up-to-date references will contribute to the up-to-date of your manuscript.

We thank the reviewer for this insightful suggestion. We have updated our list of references and the revised version now features three recent articles based on computer or mathematical models of Up-Down regimes in neuronal networks (Cakan et al, 2021; Torao-Angosto et al, 2021 and Tartaglia and Brunel, 2017). We also added short related discussion pertaining to perspectives in the discussion section.

***Changes made:***

- New recent articles related to computer or mathematical models of Up-Down regimes in neuronal networks are now cited

- Line 475: these papers are now cited to motivate perspective research

6- Please add a description for each MATLAB/Python code. It might be better to clarify which code is used in each manuscript section.

We thank the reviewer for this interesting suggestion. We have added a description in the revised version according to these lines.

***Changes made:***

- Line 225 : “The scripts to simulate the rate model are rate simu.ipynb (simulations of the model) and rate diagram.ipynb (to find the fixed points). Likewise, the scripts for the spiking

model are IAF.ipynb (simulations of the model), fixed points with astro.ipynb (self-consistent equations to find fixed points, with astrocytes), fixed points woastro.ipynb (self-consistent equations to find fixed points, without astrocytes), stability.ipynb (stability analysis of the fixed points), as well as UD analysis.m (analysis of the Up and Down states durations), and FP analysis.m (3d nullclines to find fixed points, with astrocytes).”

7- The introduction section needs to highlight the motivational contribution of the research

We think it is important for the readership of PLoS Computational Biology that the Introduction section provide the necessary biological and modelling background for a proper overview of the field. However, we agree with the reviewer that the Introduction of the initial version lacked clear exposure of the motivation of the research, in particular in its last paragraph. Therefore, we have improved the whole text of the Introduction section and totally rewritten its last paragraph. We hope the new Introduction is better than the initial one on those aspects.

***Changes made:***

- Line 52: the last paragraph of the introduction now reads: “In spite of these significant experimental observations, the mechanism by which astrocytes modulate Up-Down cortical dynamics is still unknown. In particular, it is not understood how the modulation by astrocytes interact or rely on the other identified mechanisms of Up-Down state generation in the neurons. Here, we propose a mathematical model to explore the possible mechanisms by which astrocytes control the emergence of Up-Down dynamics of their surrounding neuronal network populations. To that aim, we extended the model proposed by Jercog et al. (2017) for Up-Down dynamics in a network of excitatory and inhibitory cortical neurons with a population of astrocytes. This provided us with theoretical tools to understand how the release of gliotransmitters by the astrocytes alters the dynamics of neuronal network towards the emergence of Up-Down dynamics.”

**Reviewer #4:**

Thank you for a well written manuscript with an excellent approach and design.

We thank the reviewer this very positive appreciation of our article. We have taken into account all of his/her points of concern in the revised version.

1.my major concern is the lack of discussion how the system adjust to the biology data. network synchronization has ben described for decades and now we have a better model, however, the discussion could be a lot better

We understand the point of the reviewer. A main element of answer is that, of course, our three-population system provides information on the influence of the third population (the astrocytes) that cannot be obtained with earlier ones, that did not account for the effect of astrocytes. This is the main contribution of our new model, and the main justification for its novelty. We have tried to make this clearer in the revised version, by adding a new paragraph on this point.

***Changes made:***

- Line 437: “In our numerical simulations, the addition of gliotransmission from astrocytes was sufficient to transform a neural network prepared in the Down, silent state into a dynamical regime of spontaneous alternations between Up and Down states. The inclusion of astrocytes in our model therefore provided us with the opportunity to explore how astrocytes alter the dynamics of the neuronal firing rates in a way that switches them to the Up-Down alternation regime. A major conclusion from our model is that gliotransmission probably does not have a drastic effect on the underlying dynamics of the network. Adding gliotransmission does not modify the number nor the type of the observed bifurcations, it only alters the values of the parameters at which these bifurcations occur. As a result, gliotransmission can transform a silent neural network model into a network exhibiting Up-Down dynamics, with no change of the neuron-related parameters, and no alterations of the neural mechanisms that control the transitions between Up and Down phases. Moreover, our model suggests that the fundamental differences of signal integration in neurons versus astrocytes may be crucial in the emergence of Up-Doc regimes. In particular, the signaling delay in our spiking network model was kept three orders of magnitude larger in astrocytes compared to neurons i.e., seconds versus milliseconds. This difference of timescales turned out to be crucial for the network dynamics illustrated in fig. 5 where a stationary background of astrocytic gliotransmission events triggers spontaneous transitions between synchronized Up and Down phases of neuronal firing.”

2. How the parameter were selected for the analysis need a better rationale and expansion of this section

This is indeed an important point, that has been raised by other reviewers too. Actually, we did not use a specific parameter calibration methodology in the manuscript. The main reason for this is that the available experimental data exhibit quite a range of variability. For instance, in Jercog et al, 2017, the experimental quantities used in the discussion to compare with our model (Up or Down phase length, CV of the rates) vary quite a lot from one repetition of the experiment to the other. Over the seven repetitions of the same experiments which results are shown in their Figure 2A and B, the mean duration of the Up phases varies from 0.24s to 0.73s, for example. With other experimentalists, using other experimental setups, and recording on different cortical regions, the variability would probably be even larger.

Therefore, parameter optimization would at best have allowed to match one specific repetition of a given experiment but it is not clear what we would have learned from it. Instead, we have chosen to set the initial guess for the parameters to rough estimates from the literature when available. For instance, the quantification of propagation delays of calcium waves in 3D astrocytes made in Bindocci et al, 2017 sets an order of magnitude of around 1 to 2 sec for the maximal delay *d*_max_ of astrocytes. For the other parameters, we used bifurcation studies like those shown in the manuscript to locate regions of the parameter space in which model simulations are approximately in agreement with the variation range of the main experimental quantities of Jercog et al, 2017.

***Changes made:***

We have added a new subsection of the Methods section to explain the strategy we used to fix parameter values (section 2.3 “Parameter estimation”). We also have added a piece of text to the discussion paragraph dedicated to the comparison of the model results with the experimental results to highlight this fact.

- Line 189: “**2.3. Parameter estimation**. The experimental data available for parameter estimation of the two models above exhibit strong variability. In Jercog et al, 2017, for instance, the numbers used to quantify the experimental measurements (distribution of duration of Up or Down phases, CV of the firing rates) vary significantly from one repetition of the experiment to the other. Over the seven repetitions of the same experiments (their Figure 2A and B), the mean duration of the Up phases varies from 0.24s to 0.73s, for example. With other experimentalists, using other experimental setups, and recording on different cortical regions, the variability would probably be even larger. Therefore, the classical methodologies for parameter estimation would at best allow to match one specific repetition of a given experiment, where our objective here is to get a more generic overview of this system. Therefore, we have opted for an ad hoc method to set the values of the parameters. When available, we have set the initial guess for the parameters to rough estimates from the literature. For instance, the quantification of propagation delays of calcium waves in 3D astrocytes made in Bindocci et al, 2017 sets an order of magnitude of around 1 to 2 sec for the maximal delay of astrocytes $d^A_{\max}$. For the other parameters, we used bifurcation studies like those shown below to locate regions of the parameter space in which model simulations are approximately in agreement with the variation range of the main experimental quantities of Jercog et al, 2017. The results are given in table 1 and table 2.”
- Line 424 (Discussion): As explained in section 2.3, parameter values were set so that the model simulations exhibit behaviors similar to the experiments of Jercog et al, 2017. We now give a more detailed account of the match between model and data.

3. please check the numbers of inputs in page (13) 7. I think the numbers are bigger

We thank the reviewer for his/her vigilance. We have re double-checked all the numbers of this section in the revised version.

Great manuscript

Again, we are thankful for this very positive appreciation.
